# Harvesting at the Right Time: Maturity and its Effects on the Aromatic Characteristics of Cabernet Sauvignon Wine

**DOI:** 10.3390/molecules24152777

**Published:** 2019-07-30

**Authors:** Ting Zhao, Jiaying Wu, Jiangfei Meng, Pengbao Shi, Yulin Fang, Zhenwen Zhang, Xiangyu Sun

**Affiliations:** 1 College of Enology, Northwest A & F University, Viti-viniculture Engineering Technology Center of State Forestry and Grassland Administration, Shaanxi Engineering Research Center for Viti-Viniculture, Heyang Viti-viniculture Station, Yangling 712100, Shaanxi, China; 2 College of Food Science and Technology, Hebei Normal University of Science & Technology, Qinhuangdao 066600, Hebei, China

**Keywords:** Cabernet Sauvignon, sequential harvest, wine, aromatic characteristics

## Abstract

The aim of this paper was to investigate how maturity affects the aroma characteristics of Cabernet Sauvignon wine. A series of four *Vitis vinifera* cv. Cabernet Sauvignon wines were produced from grapes of different harvest dates. The berries of sequential harvest treatments showed an increase in total soluble solids and anthocyanin and a decrease in titratable acidity. Berry shriveling was observed as berry weight decreased. In the wines, anthocyanin, dry extract, alcoholic strength, and pH were enhanced with the sequential harvest, whereas polyphenol and tannin were decreased. The concentrations of volatile compounds in sequential harvests were found to be at higher levels. Isopentanol, phenylethyl alcohol, ethyl acetate, ethyl lactate, benzaldehyde, citronellol, and linalool significantly increased when harvest was delayed by one or two weeks. Through a principal component analysis, the volatile compounds and phenols characterizing each harvest date were clearly differentiated. These results suggest that sequential harvest may be an optional strategy for winemakers to produce high-quality wine.

## 1. Introduction

Aroma is one of the most important organoleptic characteristics for consumers and is a key attribute shaping wine styles. It is essential in the highly competitive market and the food industry [1,2]. Unique combinations of volatiles and the differences in their concentrations provide aromatically different and characteristic wines [3]. Among the several hundred volatile compounds identified in wines, the most important families are alcohols, esters, aldehydes, ketones, acids, terpenoids, norisoprenoids, pyrazines, and thiols [4].

The volatile profile of a wine is related not only to the fermentation process [5] but to the maturity of the grape as well. Some studies have focused on the evolution of volatile compounds in wines at different grape maturity levels [2,6,7,8]. Cordonnier and Bayonne emphasized the importance of harvesting during the correct stage; harvesting too early could result in a pronounced unpleasant grassy character, whereas harvesting too late could result in a loss of aroma [9]. The harvest date can have a direct influence on the final wine’s 3-isobutyl-2-methoxypyrazine (IBMP) concentrations; IBMP concentrations in wine from consecutive harvests significantly decrease [10,11]. Wines produced from Garnacha grapes with delayed harvesting contain less *β*-violet and leaf alcohols (*cis*-3-hexene-1-ol), as well as a greater amount of damascenone and geraniol [12].

Fruit maturity is among the major factors affecting secondary metabolite accumulations and determining varietal characteristics [13,14]. During grape maturation, sugar, amino acid, phenols, and potassium levels increase, while the organic acid content decreases, particularly malic acid [15,16,17]. Grape is a non-climacteric fruit species and does not ripen further after harvest. There are many factors that can affect grape maturity, including the cultivar, climate, topography, and seasonal weather conditions, and the harvest date needs to be carefully considered. Harvest dates are based on subjective evaluations of optimal fruit composition in view of the ultimate wine quality, and they may also depend on commercial targets, market constraints, processing capacity, and other factors [7,17]. Winery experience shows that wine with a positive aroma and a higher or more intense purple hue is obtained using more mature grapes [7,14,18].

Traditionally, the harvest time is simply governed by the juice sugar content and the sugar-acid ratio [3]. However, grapes are likely to face more frequent issues in warm climate viticulture, namely, prematurely reaching technical maturity [17]. Aroma and flavor potential are not yet fully developed when the grape reaches sugar maturity [11,19,20]. Besides, due to the restrictions of winery equipment, the ripening berries cannot be harvested uniformly in many areas where a single cultivar is planted. Understanding how various volatile compounds accumulate during sequential harvest is critically important. Therefore, by presenting a case study of the temporal changes in wine volatile compositions during sequential harvesting, and to optimize the harvest date to achieve optimal flavor, it is hoped that this study will illuminate the relation of sequential harvest to wine quality to aid high-quality wine production in warm-climate viticulture.

## 2. Results and Discussion

### 2.1. Normal Maturity Index

The general composition of the berries is shown in Table 1, and a good ripeness was achieved when we began to sample. Through the sequential harvesting process, berries commenced shriveling, and berry weight and length continually decreased. An increase in the sugar content of grapes can also be achieved via dehydration of grapes [21]. In this study, the increase in the grape total soluble solids (TSS) concentration was continuous from 21.83% (control berries, CK, harvest date was September 25) to 23.47% (Treatment 3, T3, harvest date was October 17). Significant promotion of the accumulation of anthocyanins was observed in sequential harvest treatments concomitant with a decrease in total tannins. Under treatment T3, the polyphenols concentration was significantly lower than those under the other treatments.

These changes may be attributed to water removal from the grape with sequential harvest. Increased transpiration and decreased phloem can result in berry shriveling concomitant with increased TSS concentrations [22,23,24]. In this study, the grape juice TSS increased and titratable acidity decreased during the sequential harvest period in accordance with other reports about the tendency of glycolic and acid during the grape ripening process [2,25]. Anthocyanin accumulations in the grape berry are correlated with increased sugar accumulation, and increased grape anthocyanins can affect wine color and aging capacity [14].

### 2.2. Enological Parameters

The enological parameters are shown in Table 2. Wine titratable acidity decreased during the sequential harvesting and was associated with an increase in pH. The pH did not exceed 4.1 by the final sampling date. The alcoholic strength increased from 11.86% vol (CK) to 12.46% vol (T2, harvest date was October 10), and the dry extract increased from 27.41 g/L to 29.49 g/L throughout the sequential harvesting period. There were no clear trends in the reducing sugar and volatile acidity of the wines with grape maturity.

An increase in the anthocyanin level was observed in the sequential harvesting treatments. The change in wine tannin concentrations showed a close relationship with the trend of the grape tannin. Work by Ristic demonstrated a strong relationship between the grape skin tannin concentration and wine tannin concentrations [26]. Moreover, the negative correlation between the change in the anthocyanins and tannins may be attributed to the capacity of anthocyanins to bind tannin under the vinification condition [27]. Interestingly, the change in wine polyphenols was attributed to the grape polyphenols; the T1 treatment had the highest polyphenols, and the wine and grape polyphenol concentrations decreased with harvesting period prolongation. Lower anthocyanin, polyphenol, and tannin levels as a result of berry shriveling during sequential harvesting were previously reported in Shiraz wines [28].

### 2.3. Qualitative and Quantitative Analyses of Volatile Compounds

A total of 47 free volatile compounds was identified and quantified in all wines, including 15 higher alcohols, 16 esters, 6 fatty acids, 6 aldehydes and ketones, and 4 terpenes and norisoprenoids. Table 3 and Table 4 show the quantitative results and odor activity values (OAVs) of these compounds. The odor descriptors and thresholds were obtained from the literature [29,30,31,32,33,34]. The most abundant volatile compounds were higher alcohols and esters, especially the higher alcohols, which made up 86%–89% of the aroma concentrations. The trace compounds were terpenes and norisoprenoids.

#### 2.3.1. Higher Alcohols

Fifteen higher alcohols were detected in this study. As shown in Table 3, higher alcohols were the largest group in terms of the aromatic compound concentrations identified in all the wine samples. They accounted for > 86% of the total volatile compounds; however, their concentrations were much lower than their thresholds, with only 1-octen-3-ol, isopentanol, and phenylethyl alcohol above the threshold (Table 4). Sequential harvesting treatments presented significantly more higher alcohols, in line with the result of a previous study [7]. Higher alcohols can contribute to a positive effect on wine aroma when they are present at less than 400 mg/L [30]. The higher alcohol concentrations in all the wine samples in this study were above 500 mg/L, which might explain the lack of desirable complexity in the aroma of wines from this wine region.

The compounds 1-Hexanol, (*E*)-3-hexen-1-ol, and (*Z*)-3-hexen-1-ol belong to the C_6_ compounds, which have a green flavor [35]. The compounds 1-Hexanol and (*E*)-3-hexen-1-ol were significantly increased in the T2 and T3 wines. Further, (*Z*)-3-hexen-1-ol has been deemed to negatively impact flavor in Cabernet Sauvignon [36]. In this study, no differences in (*Z*)-3-hexen-1-ol concentrations were found between each wine. Notably, the C_6_ alcohol (1-hexanol, (*E*)-3-hexen-1-ol, and (*Z*)-3-hexen-1-ol) concentrations decreased in T3; these changes may have been associated with shriveled grapes, as wines made from shriveled grapes contain less C_6_ alcohols [28].

Sequential harvesting treatments presented significantly higher alcohol levels due to the presence of isopentanol and phenylethyl alcohol. As their concentrations exceeded the threshold, the floral, chemical aromas from the wines were influenced. The compound 1-octen-3-ol is a well-known compound associated with a fresh mushroom odor in grapes and wines [37] and showed no notable fluctuation among all wines. Other higher alcohols, such as 4-methyl-1-pentanol, 3-methyl-1-pentanol, 2-heptanol, 1-octanol, 1-nonanol, 2-nonanol, and 1-decanol, were far lower than their threshold levels. In addition, sequential harvesting treatment can enhance the 1-heptanol concentration, which can impart a fruity aroma to the wines.

#### 2.3.2. Esters

Esters, as the most important odorants in wines, impart abundant floral and tropical fruity aromas [38]. Sixteen esters were detected in the wine samples, including 8 ethyl esters, 4 acetates esters, and 4 other esters (Table 3). The T1 and T2 treatments significantly enhanced the ester concentration.

Ethyl esters are important esters in wines. Most ethyl esters have quite low thresholds (Table 3). In this study, ethyl lactate and ethyl hexanoate were the main ethyl esters in terms of concentration, and they can impart a pleasant fruity aroma. Ethyl hexanoate and ethyl octanoate might contribute to the wine aroma directly due to their relatively high OAVs (Table 4). Compared with the CK wines, the ethyl hexanoate, ethyl heptanoate, ethyl lactate, ethyl laurate, and ethyl phenylacetate levels were significantly enhanced under the T2 treatment.

Ethyl acetate and isoamyl acetate were the main acetate esters, and the concentrations all exceeded their thresholds. The T1 and T2 treatments increased the two acetate ester concentrations. Ethyl acetate was the most abundant ester in this fraction, generating ethereal fruity aromas in the wines. A continuous increase in ethyl acetate concentration in Cabernet Sauvignon wines with grapes maturity was observed by Bindon [7]. Unlike those of ethyl acetate and isoamyl acetate, the concentrations of hexyl acetate and 2-phenethyl acetate were below their thresholds. The T2 treatment increased the 2-phenethyl acetate concentration. Notably, the isoamyl acetate and 2-phenethyl acetate concentrations in the T3 wine were inferior to those of the other treatments; isoamyl acetate and 2-phenethyl acetate can contribute to the pleasant fruity and floral aromas of the wine. This effect maybe be associated with berry over-ripening.

In addition, four other esters, methyl octanoate, methyl salicylate, butyl butanoate, and isoamyl hexanoate, were also detected in all wines (Table 3). The thresholds of methyl salicylate and butyl butanoate were unavailable in the literature, so their real impact on the wine aroma is unknown. Methyl salicylate and isoamyl hexanoate were present at levels below their thresholds (Table 3).

#### 2.3.3. Fatty Acids

Six fatty acids were detected in the wine samples (Table 3). The fatty acid concentrations increased with sequential harvesting. T2 and T3 wines presented high concentration of all fatty acids (> 8mg/L). Butanoic acid, hexanoic acid, octanoic acid, heptanoic acid *n*-decanoic acid, and 2-methyl-propanoic acid all belong to the C_6_–C_10_ fatty acids, which are important in aromatic compound balance. C_6_–C_10_ fatty acids are related to negative flavors, an unpleasant fatty odor, and even a rancid smell in wine when present at higher concentrations (>20 mg/L); however, they provide the smell of cheese and cream at concentrations of 4 to 10 mg/L [39]. As seen in Table 3, all wines had an appropriate fatty acid content, less than 10 mg/L, which can contribute a pleasant fatty smell. The 2-Methyl-propanoic acid was markedly the most abundant fatty acid, which had an OAV above 1 (Table 4). The concentration of butanoic acid increased during sequential harvesting, being present at a higher level, but its threshold was unavailable in the literature. Among the other fatty acids detected in our work, only octanoic acid was slightly above its threshold (Table 4).

#### 2.3.4. Aldehydes and Ketones

Five aldehydes (hexanal, nonanal, decanal, benzaldehyde, and benzeneacetaldehyde) and one ketone (acetoin) were identified in this study (Table 3). They can be reduced to the corresponding alcohols during the fermentation process. Compared to in the CK, the total aldehyde and ketone contents in sequential harvest wines increased. Among this group of compounds, hexanal was the only compound with a concentration exceeding its threshold, and an increase of hexanal was observed in the T2 wines. Benzaldehyde showed the highest fraction; however, the perception threshold of benzaldehydes was very high compared to its concentration. The only ketone in the wine samples was acetoin, and no notable difference was found during sequential harvesting.

#### 2.3.5. Terpenes and Norisoprenoids

Terpenes and norisoprenoids are generally associated with floral, sweet fruit and citric aromas. Norisoprenoids are trace compounds in wine, while their olfactory threshold is very low—between 0.05 and 0.09 μg/L. They usually have an odor activity [38]. Three terpenes and one norisoprenoid were detected in the wine samples, including citronellol, linalool, geraniol, and *β*-damascenone, with relatively low thresholds. Although wines have a low content of these compounds, they may contribute to the wine aroma directly (Table 3). The citronellol and linalool concentrations tended to increase, whereas the geraniol concentration did not significantly change with sequential harvesting. As seen in Table 3, citronellol levels significantly increased under sequential harvesting treatments. Linalool and *β*-damascenone were the only two of these compounds with OAVs above 1 (Table 4). Due to having the highest OAV, *β*-damascenone had a significant contribution to the wine aroma. A significant decrease was observed in *β*-damascenone during sequential harvesting.

### 2.4. Odor Activity Values (OAVs) and Aroma Profiles

**Figure 1:**
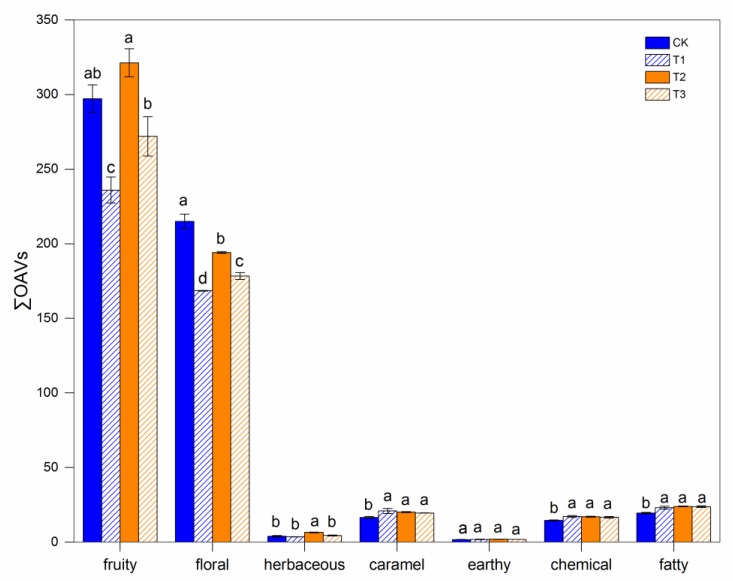
Total OAVs (∑OAV) of aroma series in wines produced from control berries (CK) and sequential harvest berries (T2, T3, and T4).

According to the report by Cai, the aromatic compounds were grouped into nine aroma series based on similar odor descriptors (Table 3) [40]. The total OAVs (∑OAV) of each series were calculated (Figure 1). The analysis of the aroma series indicated that the main aroma profiles of Cabernet Sauvignon wines in this study were fruity and floral aromas (∑OAV > 160). The earth and herbaceous series had a relatively low contribution to the overall wine aroma (∑OAV < 5).

The fruity and floral series were the major aroma series. Further, *β*-damascenone, ethyl hexanoate, isoamyl acetate, ethyl octanoate, ethyl acetate, and phenylethyl alcohol were the main contributors to the fruity and floral series, as the OAVs of these compounds all exceeded 1 (Table 4). T2 wines presented the fruitiest aromas, while T1 and T3 wines had relatively little fruity and floral aromas due to the concentrations of *β*-damascenone. For the sequential harvesting treatments, an increase was found in other aroma series (caramel, chemical, and fatty) compared with CK. The caramel, chemical, and fatty series were dominated by ispentanol. In addition, the herbaceous series was mainly based on the C_6_ compounds, including 1-hexanol, (*E*)-3-hexen-1-ol, (*Z*)-3-hexen-1-ol, and hexanal. In this study, only hexanal exceeded its threshold and was the main contributor to this series.

In addition, some volatile compounds might be present at sub-threshold concentrations; their potential contribution to the wine aroma because of additive effects should not be excluded.

### 2.5. Principal Component Analysis (PCA)

**Figure 2:**
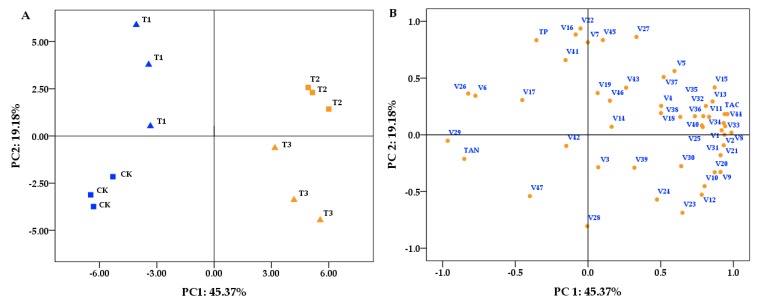
Principal Component 1 (PC1) versus PC2 scatter of the Cabernet Sauvignon wines. (**A**) Score plot. (**B**) Loading plot of Components 1 and 2. The number of volatile compounds (b) corresponds to the volatile compound number in Table 3.

To interpret the influence of sequential harvesting on the wine compound profiles, the effective data were also processed using principal component analysis (PCA). Figure 2 shows the compound loadings (Figure 2B) and the wine sample distributions (Figure 2A) across the first two principle components (PCs). The first two PCs explained 64.55% of the total variance, comprising 45.37% from PC1 and 19.18% from PC2, and it was clear that PC1 was the major discriminator in explaining the variance among the wine samples. Most of the volatile compounds, anthocyanin, and tannin contribute to the PC1 loading, which suggests that these compounds are more easily influenced by sequential harvesting. As shown in Figure 2A, CK and T1 present negative scores in PC1, and T2 and T3 lie in the first and fourth quadrants, respectively, which showed that there were major differences between CK and T2 or T3 in volatile compounds related to PC1. Only a few volatile compounds and polyphenol were observed to make contributions to the PC2 loading, and CK and T1 showed significant differences in compounds related to PC2. According to the PCA, the volatile compounds and phenols characterizing each harvest date were clearly differentiated. It could be deduced that sequential harvesting can enhance the volatile compound and anthocyanin concentrations in wine. In addition, when combined with the enological parameters, T2 was superior to T3 in avoiding excessive pH and reducing sugars in the wines.

## 3. Materials and Methods

### 3.1. Plant Material and Field Trial

*Vitis vinifera* L. cv. Cabernet Sauvignon grapes were sourced from a commercial vineyard located in Xiangfen County, Shanxi Province, China (35.7°–36.1°N, 111.1°–111.7°E, temperate continental monsoon climate) during the 2016 vintage. The vines were planted in 2012. The training system of the vines was a single cordon positioning system. Spacing within and between the vines rows was 1.0 and 3.0 m, respectively. The vines were selected on the basis of vine uniformity; the vertical shoot-positioned canopies were uniformly managed.

According to the results of the fruit maturity monitoring to determine the normal maturity of Cabernet Sauvignon, a first sampling was conducted, followed by a sampling every 7 days for a total of 4 times. The four harvest dates were September 25 (CK) and October 3 (T1), October 10 (T2), and October 17 (T3), 2016, with CK being the normal harvest date at the vineyard. To obtain representative samples, the experimental region was divided into four regions randomly arranged as a sample block. Each region was divided into three blocks, and 120 vines were planted in each block.

### 3.2. Sample Collection and Analysis of the General Index

To obtain a representative sample, for each sampling, 500 berries were randomly selected from each sample block. Grape berries were manually collected from both the inside and outside of the vine canopies included in the experiment. All samples were placed in a foam box with ice and immediately placed in a −40 °C cryogenic refrigerator after transport to the laboratory.

A total of 100 berries were randomly collected and measured for berry weight and berry length. Then, these berries were manually crushed to obtain must, and the supernatant of the must was used to measure the total soluble solids (TSS) and titratable acidity. The TSS were measured using a TD-45 digital refractometer (TOP, Zhejiang, China), and the titratable acidity was determined using sodium hydroxide titration with 0.05 M NaOH to pH 8.2. Phenolic extraction was performed using 200 berry skins. The skins were ground under liquid nitrogen protection, then the powder of the skins was lyophilized using an FD5-series Vacuum Freeze Drying Plant (GOLD-SIM, USA). Polyphenols, tannins, and anthocyanins were extracted and measured as described in a previous article [41].

### 3.3. Small-Scale Wine Making

For each replicate, approximately 80 kg of grapes was manually harvested in each harvesting period. Wines were produced by utilizing the same vinification process as in previous articles [29,42]. Briefly, three 80 kg replicates of grapes harvested at every period were directly crushed. The fermentation was conducted in stainless steel fermenters (50 L), in which 80 mL of 6% sulfurous acid was added to achieve 60 mg/L SO_2_. The volume of must was 80% of the fermenter volumes. A total of 1.6 g pectinase (Lallzyme Ex) was added to achieve 20 mg/L and mixed by hand. After maceration of the musts for 12 h, 200 mg/L of dried active yeast (*Saccharomyces cerevisiae* strain RC212, Lavlin, France) with 5% sugar water activated at 37 °C for 30 min was added to the musts according to commercial specifications. The temperature and specific gravity were monitored daily after the fermentation process was started. Alcoholic fermentation was conducted at 25–27 °C. The wines were separated from the pomace when the specific gravity decreased to 1.000 and fermentation (specific gravity 0.992–0.996) was continued to dryness (reducing sugars <2 g/L). At the end of alcoholic fermentation, 60 mg/L sulfurous acid was added to the wine to maintain 50 mg/L SO_2_. Finally, finished wines did not undergo malolactic fermentation and were bottled for analysis after two months. Enological parameters, such as alcoholic strength, reducing sugars, titratable acidity, and volatile acidity, were analyzed; for the procedures used in each index, we referred to the International Organization of Vine and Wine (OIV) standard [43].

### 3.4. Headspace Solid-Phase Microextraction (HS-SPME)

Volatile compounds in all the wine samples were extracted using headspace solid-phase microextraction (HS-SPME). A 5 mL wine sample and 1 g of NaCl were placed in a 15 mL sample vial, which contained a magnetic stirrer (1 cm) and 10 μL internal standard 4-methyl-2-pentylalcohol (1.0018 g/L, Sigma-Aldrich, Milwaukee, WI, USA). The vial was tightly capped with a polytetrafluoroethylene (PTFE) -silicon septum, heated at 40 °C for 30 min on a heating platform, and agitated at 400 rpm. The solid-phase microextraction (SPME) (50/30-μm DVB/Carboxen/PDMS, Supelco, Bellefonte, PA, USA), preconditioned according to the manufacturer’s instructions, was then inserted into the headspace, where extraction was allowed to occur for 30 min with continued heating and agitation via a magnetic stirrer. The volatiles from the fiber were subsequently desorbed by injecting the fiber into the gas chromatography (GC) injector for 8 min [44].

### 3.5. GC-MS Analysis

Gas chromatographic analyses were performed with an Agilent gas chromatograph model 7890 equipped using an Agilent 5975 mass spectrometer and 7683 automatic sampler (Agilent, Santa Clara, CA, USA). Samples were separated on an HP-INNOWAX capillary column (60 m × 0.25 mm × 0.25 μm, J &W Scientific, Folsom, CA, USA). The carrier gas was helium (purity > 99.999%) at 1 mL/min. The temperature in the injection port was 250 °C. Samples were injected by placing the SPME fiber at the GC inlet for 8 min in the splitless mode. The oven temperature program was as follows: 50 °C for 1 min, then increased to 220 °C at a rate of 3 °C/min and held at 220 °C for 5 min. The mass detector conditions were as follows: electron impact mode (MS/EI) at 70 eV, mass scanning range *m*/*z* 20 to 350 U, ionic source temperature 230 °C. The mass spectrometry interface temperature was 280 °C. The mass spectrophotometer was operated in the selective ion mode under autotune conditions, and the area of each peak was determined using the ChemStation software F.01.01.2317 (Agilent Technologies, Inc. Santa Clara, CA, USA) [44].

### 3.6. Volatile Compound Identification and Quantification

A synthetic wine matrix was prepared in distilled water containing 13% ethanol (*v*/*v*), 2 g/L glucose, and 5 g/L citric acid. The pH was adjusted to 3.8 using a 5 M NaOH solution. All aromatic compound standards with purity greater than 99% were purchased from Sigma-Aldrich (Milwaukee, WI, USA). The volatile compounds stock solution was prepared and dissolved in the synthetic wine matrix. The standard solution was successively diluted to fifteen levels, and 10 μL internal standard (4-methyl-2-pentylalcohol, 1.0018 g/L) was added. Afterwards, the known concentrations of the standard volatile compounds were extracted and analyzed under the same conditions as the wine samples.

The volatile compound identification and quantification methods were based on a previous work [44]. Volatile compounds were identified by a comparison of Kováts’ retention indices based on the even *n*-alkanes (C7-C24) (Supelco, Bellefonte, PA, USA) of the reference standard, and mass spectra matching to the standard National Institute of Standards and Technology Library (NIST11) ([45]. Comparison of retention indices to those reported in the literature was used without available external standards. For quantification, all the calibration curves had regression coefficients greater than 95%; the detailed quantification information is listed in Table 5. The volatile compound concentrations were calculated from the quantitative ion peak areas with regard to the internal standard. The compounds without established calibration curves were quantified according to the standards with the same functional group or similar numbers of carbon atoms.

### 3.7. Odor Activity Values (OAVs) and Aroma Series

The odor activity value (OAV) is commonly used to evaluate the contribution of a volatile compound to a wine’s characteristic aroma [46,47]. The OAVs were calculated using the equation OAV = *c*/*t*, where *c* is the concentration (in μg/L) of each compound in the wine sample and *t* is the odor threshold value (in μg/L) of the compound in water/ethanol solution [46]. The perception threshold was found in the literature (Table 3).

To simulate the aroma profile according to the wine volatile composition, the volatile compounds were grouped based on similar odor descriptors. Then, the sum of the OAVs (∑OAV) was calculated, which simulated the wine aroma profile. In this study, the volatile compounds were grouped into nine aroma series, namely, fruity, floral, herbaceous, nutty, caramel, earthy, chemical, fatty, and roasted. The aroma series division followed one performed in a previous study [40]. Due to the complexity of aroma characteristics, some volatile compounds may be included in several aroma series.

### 3.8. Statistical Analysis

Statistical data processing was performed using the software Statistical Product and Service Solutions (SPSS 20.0) for Windows (IBM, Armonk, NY, USA). Statistical analyses of the data were performed using one-way analysis of variance (ANOVA) and Duncan’s test at the *p* < 0.05 level. To obtain an overview of the different wine samples, the data of volatile compounds and phenols were subjected to principle component analysis (PCA) to visualize all information in the data set. All plots were prepared using Origin 2016 (OriginLab Corporation, Northampton, MA, USA).

## 4. Conclusions

This work investigated the effect of sequential harvesting on the volatile profiles of Cabernet Sauvignon wines. We found that sequential harvesting treatments enhanced the TSS and anthocyanin levels in the berries, while the titratable acid levels decreased. Berry shriveling was observed with over-ripening and resulted in decreased berry weight. Meanwhile, enological parameters and wine volatile compounds were influenced by the sequential harvesting treatments. Volatile profiles, anthocyanins, dry extracts, alcoholic strength, and pH increased under sequential harvesting. The volatile profiles in the sequential harvesting treatments were found to be more abundant, especially in the T1 and T2 wines; isopentanol, phenylethyl alcohol, ethyl acetate, ethyl lactate, benzaldehyde, citronellol, and linalool all showed higher levels. The fruity aroma was enhanced, while the level of *β*-damascenone was lessened in the T2 wines. The PCA indicated that sequential harvesting could enhance the concentrations of volatile compounds in wine. In conclusion, sequential harvesting is an optional strategy for winemakers to avoid the restrictions of winery equipment when berries reach maturity in large areas growing a single wine grape cultivar.

## Figures and Tables

**Table 1:** General composition of Cabernet Sauvignon grapes in the control berries and sequential harvest berries.

Parameters	Treatments			
	CK	T1	T2	T3
Berry weight (g)	1.33 ± 0.02a	1.31 ± 0.03a	1.26 ± 0.02b	1.21 ± 0.03c
Berry length (mm)	13.07 ± 0.83a	12.34 ± 1.25b	12.26 ± 0.91b	12.19 ± 0.94b
Total soluble solid (%)	21.8 ± 0.03d	22.7 ± 0.17c	23.0 ± 0.06b	23.5 ± 0.03a
Titratable acidity (g/L tartaric acid)	4.48 ± 0.12a	4.10 ± 0.06b	4.01 ± 0.10b	3.83 ± 0.05b
Total anthocyanins (mg ME/g)	9.91 ± 0.04c	10.89 ± 0.18b	11.21 ± 0.23ab	11.68 ± 0.16a
Total tannins (mg CE/g)	29.07 ± 1.04a	26.00 ± 0.87a	23.56 ± 0.82b	23.81 ± 0.33b
Total polyphenols (mg GAE/g)	41.61 ± 0.69a	42.15 ± 0.65a	39.85 ± 0.60ab	38.43 ± 0.98b

Titratable acidity is expressed in g/L of tartaric acid. The total anthocyanin, total polyphenol, and total tannin concentrations are expressed in mg/g cyanidin-3-mono-glucoside, mg/g gallic acid equivalence, and mg/g (+)-catechin per gram of dry berry skin, respectively. The four harvest dates were September 25 (CK) and October 3 (T1), October 10 (T2), and October 17 (T3), 2016, with CK being the normal harvest date at the vineyard.

**Table 2:** General enological parameters composition of Cabernet Sauvignon wines made from control berries and sequential harvest berries.

Parameters	Treatment			
	CK	T1	T2	T3
Reducing sugars (g/L)	2.28 ± 0.10b	2.79 ± 0.08a	2.27 ± 0.05b	2.75 ± 0.10a
Titratable acidity (g/L)	5.72 ± 0.01a	5.32 ± 0.01b	5.16 ± 0.01c	5.05 ± 0.00d
pH	3.94 ± 0.00d	4.01 ± 0.00c	4.05 ± 0.01b	4.07 ± 0.01a
Alcoholic strength by volume (% vol)	11.86 ± 0.08c	12.39 ± 0.07b	12.46 ± 0.03a	12.41 ± 0.04a
Dry extract (g/L)	27.41 ± 0.23b	27.52 ± 0.14b	29.47 ± 0.08a	29.49 ± 0.16a
Volatile acidity (g/L)	0.43 ± 0.00b	0.46 ± 0.00a	0.42 ± 0.00b	0.39 ± 0.00c
Total anthocyanins (mg/L)	89.59 ± 4.74d	104.00 ± 2.04c	123.48 ± 1.32a	120.09 ± 1.34b
Total polyphenols (mg/L)	1279.28 ± 12.27b	1458.02 ± 21.23a	1338.86 ± 21.92b	1208.88 ± 29.99c
Total tannins (mg/L)	494.70 ± 26.48a	412.06 ± 14.68b	343.52 ± 8.71c	345.07 ± 22.34c

The results are expressed as the mean values ± standard deviation of the triplicate samples. Reducing sugar is expressed in g/L glucose, titratable acidity in g/L tartaric acid, and volatile acidity in g/L acetic acid. Total anthocyanins, total polyphenols and total tannins are expressed in mg/L cyanidin-3-mono-glucoside, mg/L gallic acid, and mg/L ( + )-catechin, respectively.

**Table 3:** Concentrations (μg/L, mean ± standard deviation) of free volatile compounds in Cabernet Sauvignon wines produced from control berries (CK) and sequential harvest berries (T1, T2, and T3).

No.	Volatile Aroma Compounds	Treatments				Descriptor	Odor Threshold	Aroma Series ^a^
		CK	T1	T2	T3		(μg/L)	
***Higher Alcohols***								
1	1-Hexanol	5502.53 ± 45.96c	5576.00 ± 204.51c	9670.76 ± 244.94a	7590.31 ± 340.23b	Green, grass [28,29]	8000 [28,29,30,31]	3
2	(*E*)-3-Hexen-1-ol	91.42 ± 0.25c	94.35 ± 3.63c	203.4 ± 9.67a	155.24 ± 8.79b	Green, floral [28,29]	400 [28,29,30]	3
3	(*Z*)-3-Hexen-1-ol	147.81 ± 0.87a	139.49 ± 9.74a	145.35 ± 2.82a	138.59 ± 11.96a	Green [28,29]	400 [28,29,30]	3, 8
4	1-Octen-3-ol	33.21 ± 0.20a	34.70 ± 2.17a	37.10 ± 2.20a	34.46 ± 0.49a	Mushroom [33]	20 [33]	6
5	Isopentanol	435327.94 ± 9826.01b	515279.31 ± 8921.83a	511874.25 ± 10960.07a	498725.37 ± 16620.34a	Whiskey, nail polish [29,33]	30,000 [28,29,30]	7, 5, 8
6	4-Methyl-1-pentanol	4.98 ± 0.14ab	5.4 ± 0.54a	3.89 ± 0.02c	4.37 ± 0.1bc	almond, toasty [33]	50,000 [28,31]	4, 5, 9
7	3-Methyl-1-pentanol	371.49 ± 8.78b	476 ± 30.32a	423.45 ± 8.27ab	376.18 ± 15.96b	vinous, herbaceous, cacao [33]	50,000 [31]	3, 7
8	1-Heptanol	539.16 ± 5.48c	841.82 ± 19.44b	1432.52 ± 30a	1450.37 ± 71.85a	Grape, sweet [28,29]	1000 [28,29]	1
9	2-Heptanol	6.23 ± 0.23b	6.05 ± 0.22b	8.88 ± 0.52a	9.46 ± 0.28a	Fruity, moldy, musty [28]	70 [28]	1, 6
10	1-Octanol	25.56 ± 0.4b	23.75 ± 1.01b	29.16 ± 0.53a	31.89 ± 1.17a	Intense citrus, roses [28,29]	120 [28,29]	2
11	1-Nonanol	21.36 ± 0.69c	23.06 ± 0.16c	34.64 ± 0.06a	26 ± 1.54b	Green [29]	600 [29]	3
12	2-Nonanol	4.07 ± 0.08b	1.86 ± 1.01c	6.3 ± 0.19a	5.83 ± 0.29a	unpleasant floral [28]	58 [28]	-
13	(*Z*)-6-Nonen-1-ol	7.93 ± 0.1d	10.03 ± 0.02c	15.66 ± 0.23a	11.16 ± 0.65b	-	Unknown	
14	1-Decanol	4.11 ± 0.07ab	3.99 ± 0.12b	4.34 ± 0.02a	3.96 ± 0.06b	Orange flowery, special fatty [28,29]	400 [28,29]	1, 2, 7, 8
15	Phenylethyl alcohol	107270.06 ± 805.29c	134569.36 ± 2530.81b	146411.1 ± 588.25a	143031.88 ± 177.6a	Sweet rose [28,29]	14,000 [28,29]	2
	Subtotal	549357.86 ± 9069.56b	657085.16 ± 15251.97a	670300.79 ± 10666.27a	651595.07 ± 16895.79a			
***Esters***								
16	Ethyl acetate	55638.68 ± 1217.48c	73720.51 ± 2084.52a	65773.82 ± 952.74b	54064.14 ± 1882.87c	Ethereal fruity [28,29]	7500 [28,29,30,31]	1
17	Isoamyl acetate	756.79 ± 166.15a	867.13 ± 112.66a	792.31 ± 86.13a	370.43 ± 70.58b	Intense banana [28,29]	30 [28,29,30,31]	1
18	Hexyl acetate	3.23 ± 0.14a	2.22 ± 0.55a	2.25 ± 0.53a	2.39 ± 0.22a	Pleasant fruity, pear, floral [28,29]	670 [28,29]	1, 2
19	2-Phenethyl acetate	126.92 ± 0.57b	116.55 ± 0.73c	175.35 ± 1.37a	71.91 ± 1.33d	Pleasant, floral [29]	650 [29,30]	2
20	Ethyl hexanoate	288.85 ± 5.14b	202.25 ± 60.01b	519.39 ± 28.64a	427.21 ± 39.1a	Green apple, fruity, strawberry, anise [29,31]	5 [29]	1
21	Ethyl heptanoate	1.49 ± 0.03b	2.43 ± 0.41b	4.05 ± 0.34a	4.28 ± 0.4a	Pineapple, fruity [29]	220 [29]	1
22	Ethyl lactate	19942.01 ± 767.99c	32095.67 ± 1039.95a	25796.29 ± 138.13b	19743.84 ± 50.72c	Lactic, raspberry [28,32]	150,000 [32]	1, 8
23	Ethyl octanoate	30.17 ± 0.17ab	27.04 ± 2.9b	32.28 ± 1.05ab	34.46 ± 1.57a	Pineapple, pear, floral [28]	2 [28]	1, 2
24	Ethyl decanoate	91.83 ± 0.85b	91.19 ± 1.97b	91.81 ± 3.46b	114.00 ± 5.82a	Fruity, fatty, pleasant [28,29]	200 [28,29]	1
25	Ethyl laurate	30.95 ± 0.07b	31.77 ± 0.25a	32.11 ± 0.15a	32.17 ± 0.31a	Oily, fatty, fruity [28]	1500 [28]	1, 8
26	Ethyl salicylate	17.64 ± 0a	17.64 ± 0.02a	17.60 ± 0.01b	17.55 ± 0a	-	Unknown	
27	Ethyl phenylacetate	10.51 ± 0c	13.19 ± 0.04a	13.42 ± 0.05a	11.34 ± 0.33b	rose, floral [33]	250 [31]	2
28	Methyl octanoate ^b^	0.72 ± 0.01a	0.61 ± 0.61a	0.66 ± 0.04a	0.69 ± 0.05a	Intense citrus [28]	200 [28,30]	1
29	Methyl salicylate	13.49 ± 0.88a	11.48 ± 0.46b	7.31 ± 0.15c	6.76 ± 0.05c	-	40 [33]	
30	Butyl butanoate	12.85 ± 0.2a	15.22 ± 1.13a	17.34 ± 0.52a	18.97 ± 4.14a	-	Unknown	
31	Isoamyl hexanoate	2.99 ± 0.01b	3.13 ± 0.02b	3.46 ± 0.07a	3.58 ± 0.11a	Pineapple, cheese [33]	1000 [33]	1
	Subtotal	76969.13 ± 1822.77c	107214.97 ± 3154.25a	93289.11 ± 934.22b	74922.23 ± 1955.94c			
***Fatty acids***								
32	Butanoic acid	1516.75 ± 77.97b	1621.66 ± 111.64ab	1835.97 ± 21.25a	1881.57 ± 78.1a	-	Unknown	
33	Hexanoic acid ^b^	17.56 ± 0.98b	24.15 ± 2.63b	42.91 ± 1.33a	46.46 ± 2.95a	Cheese, unpleasant copra, oil odor [28]	3000 [28,31]	8
34	Heptanoic acid	55.30 ± 0c	58.98 ± 2.15b	65.14 ± 0.39a	66.91 ± 0.47a	Fatty, dry [28]	3000 [28]	8
35	Octanoic acid	854.27 ± 10.2c	913.68 ± 74.9bc	1087.27 ± 38.21ab	1181.95 ± 106.65a	Rancid, harsh, cheese, fatty acid [28,29]	500 [28,29,30]	8
36	*n*-Decanoic acid	183.69 ± 0.58a	185.22 ± 3.39a	189.44 ± 0.7a	189.8 ± 0.6a	Sour, fatty, unpleasant [29]	1000 [29,30]	8
37	2-Methyl-propanoic acid	4388.59 ± 266.85b	5425.47 ± 381.78a	5273.97 ± 132.72ab	5677.87 ± 248.70a	Fatty [28]	2300 [30]	8
	Subtotal	7016.16 ± 356.57b	8228.17 ± 194.71ab	8494.69 ± 193.21a	9044.56 ± 437.48a			
***Aldehydes and ketones***								
38	Hexanal	12.65 ± 2.51b	10.39 ± 0.4b	20.94 ± 1.7a	12.66 ± 0.77b	Intense green, grass [29]	5 [29]	3
39	Nonanal ^b^	2.00 ± 0.07a	2.37 ± 1.03a	2.44 ± 0.16a	2.75 ± 0.41a	Green, slightly pungent [29]	15 [29,30]	3
40	Decanal	0.94 ± 0.11b	0.97 ± 0.02b	1.96 ± 0.12a	1.24 ± 0.08b	Grassy, orange, skin-like [28]	10 [30]	1, 8
41	Benzaldehyde	98.85 ± 0.18d	304.07 ± 1.72a	136.91 ± 2.72c	163.13 ± 6.02b	Roasted, almond [28,29]	2000 [28,29]	9
42	Benzeneacetaldehyde ^b^	2.26 ± 0.10c	2.77 ± 0.06b	3,19 ± 0.03a	3.03 ± 0.04a	Floral, rose, honey [34]	5 [30]	2, 5
43	Acetoin	215.27 ± 2.11a	221.52 ± 4.72a	219.5 ± 1.55a	225.96 ± 7.21a	Fatty, cream [31]	150,000 [30]	8
	Subtotal	331.97 ± 4.67d	542.08 ± 5.77a	384.93 ± 2.82c	408.77 ± 0.03b			
***Terpenes and norisoprenoids***								
44	Citronellol	6.33 ± 0.02d	7.15 ± 0.04c	9.25 ± 0.14a	8.4 ± 0.05b	Fruity rosy, green lemon [28,29]	100 [28,29,30]	2
45	Linalool	25.18 ± 5.29a	63.9 ± 27.12a	44.88 ± 0.63a	42.04 ± 11.74a	Flowery, fruity, muscat [28,29]	25 [28,29]	2, 5
46	Geraniol	18.52 ± 0.71a	18.19 ± 0.53a	18.55 ± 0.72a	18.76 ± 0.82a	Citric [29]	30 [29,30]	1
47	*β*-damascenone	9.51 ± 0.25a	7.09 ± 0.02c	8.22 ± 0.01b	7.4 ± 0.1c	Honey, sweet [29]	0.05 [29,30]	1, 2, 5
	Subtotal	59.53 ± 5.73a	96.33 ± 27.63a	80.90 ± 0.22a	76.60 ± 12.41a			
	Total (mg/L)	633734.65 ± 11259.30b	773167.71 ± 19442.11a	772550.42 ± 11675.33a	736047.23 ± 18899.27a			

Note: ^a^ Aroma series 1 = fruity, 2 = floral, 3 = herbaceous (or vegetal), 4 = nutty, 5 = caramel, 6 = earthy, 7 = chemical, 8 = fatty, 9 = roasted. ^b^ The volatile compound concentrations were less than the limit of quantification (LQ). Different letters in the same row means significant differences according to Duncan test (*p* < 0.05).

**Table 4:** Odor activity values (OAVs) of main aroma compounds (OAV > 0.1) determined in the Cabernet Sauvignon wines produced from control berries (CK) and sequential harvest berries (T2, T3, and T4).

Volatile Aroma Compounds	Treatments			
	CK	T1	T2	T3
***Higher Alcohols***				
1-Hexanol	0.69 ± 0.01c	0.7 ± 0.03c	1.21 ± 0.03a	0.95 ± 0.04b
(E)-3-Hexen-1-ol	0.23 ± 0.00c	0.24 ± 0.01c	0.51 ± 0.02a	0.39 ± 0.02b
(Z)-3-Hexen-1-ol	0.37 ± 0.00a	0.35 ± 0.02a	0.36 ± 0.01a	0.35 ± 0.03a
1-Octen-3-ol	1.66 ± 0.01a	1.74 ± 0.15a	1.85 ± 0.11a	1.72 ± 0.02a
Isopentanol	14.51 ± 0.33b	17.18 ± 0.40a	17.06 ± 0.37a	16.62 ± 0.55a
1-Heptanol	0.54 ± 0.01c	0.84 ± 0.02b	1.43 ± 0.03a	1.45 ± 0.07a
2-Heptanol	0.09 ± 0.00b	0.09 ± 0.00b	0.13 ± 0.01a	0.14 ± 0.00a
1-Octanol	0.21 ± 0.00b	0.2 ± 0.01b	0.24 ± 0.00a	0.27 ± 0.01a
2-Nonanol	0.07 ± 0.00b	0.03 ± 0.02c	0.11 ± 0.00a	0.10 ± 0.00a
Phenylethyl alcohol	7.66 ± 0.06c	9.61 ± 0.18b	10.46 ± 0.04a	10.22 ± 0.01a
***Esters***				
Ethyl acetate	7.42 ± 0.16c	9.83 ± 0.28a	8.77 ± 0.13b	7.21 ± 0.25c
Isoamyl acetate	25.23 ± 5.54a	28.9 ± 3.76a	26.41 ± 2.87a	12.35 ± 2.35b
2-Phenethyl acetate	0.2 ± 0.00b	0.18 ± 0.00c	0.27 ± 0.00a	0.11 ± 0.00d
Ethyl hexanoate	57.77 ± 1.03b	40.45 ± 12b	103.88 ± 5.73a	85.44 ± 7.82a
Ethyl lactate	0.13 ± 0.01c	0.21 ± 0.01a	0.17 ± 0.00b	0.13 ± 0.00c
Ethyl octanoate	15.08 ± 0.09ab	13.52 ± 1.45b	16.14 ± 0.53ab	17.23 ± 0.78a
Ethyl decanoate	0.46 ± 0.00b	0.46 ± 0.01b	0.46 ± 0.02b	0.57 ± 0.03a
Methyl salicylate	0.34 ± 0.02a	0.29 ± 0.01b	0.18 ± 0.00c	0.17 ± 0.00c
***Fatty acids***				
Octanoic acid	1.71 ± 0.02c	1.83 ± 0.15bc	2.17 ± 0.08ab	2.36 ± 0.21a
*n*-Decanoic acid	0.18 ± 0.00a	0.19 ± 0.00a	0.19 ± 0.00a	0.19 ± 0.00a
2-Methyl-propanoic acid	1.91 ± 0.12b	2.36 ± 0.17a	2.29 ± 0.10ab	2.47 ± 0.19b
***Aldehydes and ketones***				
Hexanal	2.53 ± 0.5b	2.08 ± 0.08b	4.19 ± 0.34a	2.53 ± 0.15b
Decanal	0.09 ± 0.01b	0.1 ± 0.00b	0.2 ± 0.01a	0.12 ± 0.01b
Benzaldehyde	0.05 ± 0.00d	0.15 ± 0.00a	0.07 ± 0.00c	0.08 ± 0.00b
***Terpenes and norisoprenoids***				
Linalool	1.01 ± 0.21a	2.56 ± 1.08a	1.8 ± 0.03a	1.68 ± 0.47a
Geraniol	0.62 ± 0.02a	0.61 ± 0.02a	0.62 ± 0.02a	0.63 ± 0.03a
*β*-damascenone	190.29 ± 5.01a	141.72 ± 0.47c	164.38 ± 0.11b	148.06 ± 2.00c

**Table 5:** Calibration parameters (Chemical Abstracts Service Number (CASN), Retention Indices (RI), Identification (ID), Manufactures, Purity, Internal standards, Quantitative ion), calibration curves’ linear correlation coefficients (R^2^), and range for the quantitative analysis of volatile aroma compounds in wine using gas chromatography-mass spectrometry (GC-MS)-solid-phase microextraction (SPME).

No.	Volatile Compounds	CAS	RI ^a^	ID ^b^	Manufacturers	Purity	Internal Standards	Quantitative Ion	Calibration Curves	R^2^	Range (µg/L)
1	1-Hexanol	111273	1334	A	Sigma-Aldrich	0.98	1-Hexanol	56	Y = 3803.37X-10.73	0.97	1.26–20610
2	(*E*)-3-Hexen-1-ol	928972	1346	A	Sigma-Aldrich	0.97	(*E*)-3-Hexen-1-ol	41	Y = 7363.48X-43.64	0.96	0.75–406
3	(*Z*)-3-Hexen-1-ol	928961	1366	A	Sigma-Aldrich	0.98	(*Z*)-3-Hexen-2-ol	82	Y = 10310.57X + 3.49	0.96	0.25–1033
4	1-Octen-3-ol	3391864	1541	A	Sigma-Aldrich	0.98	1-Octen-3-ol	57	Y = 452.22X + 0.23	0.97	0.52–133
5	Isopentanol	123513	1189	A	Sigma-Aldrich	0.99	Isopentanol	55	Y = 18884.34X + 8.20	0.98	25–36440
6	4-Methyl-1-pentanol	626891	1297	B			3-Methyl-1-pentanol	82	Y = 271.24X − 0.66	0.97	3–5685
7	3-Methyl-1-pentanol	589355	1310	A	Sigma-Aldrich	0.97	3-Methyl-1-pentanol	56	Y = 6019.10X + 6.37	0.97	3–11370
8	1-Heptanol	111706	1437	A	Sigma-Aldrich	0.98	1-Heptanol	88	Y = 2106.31X + 8.31	0.97	6–732
9	2-Heptanol	543497	1303	B			1-Heptanol	45	Y = 2106.31X + 8.32	0.97	6–732
10	1-Octanol	111875	1433	A	Sigma-Aldrich	0.99	1-Octanol	56	Y = 641.39X − 2.68	0.98	1–69
11	1-Nonanol	143088	1648	B			(Z)-3-Nonen-1-ol	68	Y = 692.85X + -0.75	0.98	0.16–163
12	2-Nonanol	628999	1501	A	Sigma-Aldrich	0.99	2-Nonanol	45	Y = 80.67X − 0.37	0.99	0.08–81
13	(Z)-6-Nonen-1-ol	35854865	1706	A	Sigma-Aldrich	0.97	(Z)-6-nonen-1-ol	41	Y = 689.12X + 0.63	0.97	2–1112
14	1-Decanol	112301	1766	A	Sigma-Aldrich	0.99	1-Decanol	70	Y = 147.26X − 1.81	0.99	2–605
15	Phenylethyl alcohol	60128	1910	A	Sigma-Aldrich	0.99	Phenylethyl alcohol	91	Y = 3638.40X + 764.85	0.97	80–2569
16	Ethyl acetate	141786	745	A	Sigma-Aldrich	0.99	Ethyl acetate	71	Y = 16104.02X + 102.45	1	1336–158350
17	Isoamyl acetate	123922	1122	A	Sigma-Aldrich	0.95	Isoamyl acetate	57	Y = 299.43X-8.54	0.98	1–2888
18	Hexyl acetate	142927	1262	A	Sigma-Aldrich	0.99	Hexyl acetate	84	Y = 112.91X-7.23	0.99	1–1540
19	2-Phenethyl acetate	103457	1816	A	Sigma-Aldrich	0.99	2-Phenethyl acetate	104	Y = 130.41X + 1.40	0.94	3–116
20	Ethyl hexanoate	123660	1223	A	Sigma-Aldrich	0.99	Ethyl hexanoate	88	Y = 171.73X-11.01	0.98	3–2680
21	Ethyl heptanoate	106309	1322	A	Sigma-Aldrich	0.98	Ethyl heptanoate	88	Y = 52.65X-0.25	1	0.06–118
22	Ethyl lactate	97643	1350	A	Sigma-Aldrich	0.98	Ethyl lactate	45	Y = 73602.47X + 487.39	0.97	85–22640
23	Ethyl octanoate	106321	1429	A	Sigma-Aldrich	0.99	Ethyl octanoate	88	Y = 5.63X + 17.22	0.97	20–3227
24	Ethyl decanoate	110383	1635	A	Sigma-Aldrich	0.99	Ethyl decanoate	88	Y = 49.10X + 28.54	0.96	49–1580
25	Ethyl laurate	106332	1842	A	Sigma-Aldrich	0.98	Ethyl laurate	88	Y = 49.10X + 28.54	1	3–355
26	Ethyl salicylate	118616	1813	A	Sigma-Aldrich	0.98	Ethyl salicylate	120	Y = 64.11X + 17.39	0.99	1.33–341
27	Ethyl phenylacetate	101973	1785	A	Sigma-Aldrich	0.99	Ethyl phenylacetate	91	Y = 215.77X + 0.22	0.98	0.69–176
28	Methyl octanoate	111115	1377	A	Sigma-Aldrich	0.99	Methyl octanoate	74	Y = 32.16X + 0.06	1.00	1–138
29	Methyl salicylate	119368	1778	A	Sigma-Aldrich	0.99	Methyl salicylate	120	Y = 500.00X + 3.56	0.97	0.79–1612
30	Butyl butanoate	109217	1868	B			Ethyl butanoate	71	Y = 2819.02X-5.03	1	0.33–2740
31	Isopentyl hexanoate	2198610	1458	A	Sigma-Aldrich	0.98	Isopentyl hexanoate	70	Y = 33.39X + 2.24	0.98	0.24–500
32	Butanoic acid	107926	1612	A	Sigma-Aldrich	0.99	Butanoic acid	60	Y = 52865.86X + 119.94	0.97	31–4000
33	Hexanoic acid	142621	1837	A	Sigma-Aldrich	0.99	Hexanoic acid	60	Y = 4039.10X + 768.38	0.98	102–1625
34	Heptanoic acid	111148	1945	A	Sigma-Aldrich	0.99	Heptanoic acid	60	Y = 1856.05X + 55.30	0.94	20–635
35	Octanoic acid	124072	2053	A	Sigma-Aldrich	0.99	Octanoic acid	60	Y = 4351.97X + 40.72	0.96	141–2254
36	*n*-Decanoic acid	334485	2262	A	Sigma-Aldrich	0.99	Decanoic acid	60	Y = 1371.48X-129.36	0.99	9–1126
37	2-Methyl-propanoic acid	79312	1590	B			Butanoic acid	43	Y = 52865.86X + 119.94	0.97	31–4000
38	Hexanal	66251	1099	B			(E)-2-Hexenal,	44	Y = 2957.82X-26.15	0.98	10–2530
39	Nonanal	124196	1382	B			(E)-2-Nonenal	57	Y = 317.92X + 2.21	0.99	3–188
40	Decanal	112312	1502	A	Sigma-Aldrich	0.98	Decanal	43	Y = 156.50X-0.34	0.97	0.13–130
41	Benzaldehyde	100527	1515	A	Sigma-Aldrich	0.99	Benzaldehyde	77	Y = 561.57X + 3.66	0.99	1.5–288
42	Benzeneacetaldehyde	122781	1639	A	Sigma-Aldrich	0.99	Benzeneacetaldehyde	91	Y = 5800.28X-0.77	0.94	13–831
43	Acetoin	513860	1298	A	Sigma-Aldrich	0.96	Acetoin	45	Y = 3211.64X + 180.55	0.95	211–59467
44	Citronellol	106229	1757	A	Sigma-Aldrich	0.95	Citronellol	41	Y = 348.91X + 0.28	0.99	1–66
45	Linalool	78706	1532	A	Sigma-Aldrich	0.97	Linalool	71	Y = 119.55X-0.07	0.98	0.03–31
46	Geraniol	106241	1780	A	Sigma-Aldrich	0.99	Geraniol	69	Y = 503.61X + 17.29	0.98	2.77–355
47	*β*-damascenone	23726934	1823	A	Sigma-Aldrich	0.98	(E)-*β*-damascenone	177	Y = 130.17X + 0.34	1	0.57–580

Note: ^a^ The retention index (RI) was calculated on the HP-INNOWAX capillary column. ^b^ In identification of the compounds, “A” means those identified by mass spectrum and RI agree with standards, “B” means those tentatively identified by mass spectrum agree with the mass spectral database and RI agrees with literature.

## References

[1] Selli S., Cabaroglu T., Canbas A., Erten H., Nurgel C., Lepoutre J.P., Gunata Z. (2004). Volatile composition of red wine from cv. Kalecik Karasι grown in central Anatolia. Food Chem..

[2] Yang C., Wang Y., Wu B., Fang J., Li S. (2011). Volatile compounds evolution of three table grapes with different flavour during and after maturation. Food Chem..

[3] Ribéreau-Gayon P., Glories Y., Maujean A., Dubourdieu D. (2006). Handbook of Enology: Volume 2. The Chemistry of Wine Stabilization and Treatments.

[4] Gonzalez-Barreiro C., Rial-Otero R., Cancho-Grande B., Simal-Gandara J. (2015). Wine aroma compounds in grapes: A critical review. Crit. Rev. Food Sci. Nutr..

[5] Styger G., Prior B., Bauer F. (2011). Wine flavor and aroma. J. Ind. Microbiol. Biotechnol..

[6] Bindon K., Varela C., Kennedy J., Holt H., Herderich M. (2013). Relationships between harvest time and wine composition in *Vitis vinifera* L. cv. Cabernet Sauvignon 1. Grape and wine chemistry. Food Chem..

[7] Fenoll J., Manso A., Hellín P., Ruiz L., Flores P. (2009). Changes in the aromatic composition of the *Vitis vinifera* grape Muscat Hamburg during ripening. Food Chem..

[8] Park S.K., Morrison J.C., Adams D.O., Noble A.C. (1991). Distribution of free and glycosidically bound monoterpenes in the skin and mesocarp of Muscat of Alexandria grapes during development. J. Agric. Food Chem..

[9] Cordonnier R., Bayonne C. (1978). Les composantes varietales et prefermentaires de l’ arome des vins. Rev. Fr. Doenol..

[10] Boubée D.R.D., Leeuwen C.V., Dubourdieu D. (2000). Organoleptic impact of 2-methoxy-3-isobutylpyrazine on red Bordeaux and Loire wines. Effect of environmental conditions on concentrations in grapes during ripening. J. Agric. Food Chem..

[11] Schelezki O.J., Suklje K., Boss P.K., Jeffery D.W. (2018). Comparison of consecutive harvests versus blending treatments to produce lower alcohol wines from Cabernet Sauvignon grapes: Impact on wine volatile composition and sensory properties. Food Chem..

[12] Sabon I., De R.G., Kotseridis Y., Bertrand A. (2002). Determination of volatile compounds in Grenache wines in relation with different terroirs in the Rhone Valley. J. Agric. Food Chem..

[13] Gomez E., Martinez A., Laencina J. (1995). Changes in volatile compounds during maturation of some grape varieties. J. Sci. Food Agric..

[14] Pérez-Magariño S., González-San José M.L. (2006). Polyphenols and colour variability of red wines made from grapes harvested at different ripeness grade. Food Chem..

[15] Adams D.O. (2006). Phenolics and ripening in grape berries. Am. J. Enol. Vitic..

[16] Ollat N., Diakou-Verdin P., Carde J.P., Barrieu F., Gaudillère J.P., Moing A. (2002). Grape berry development: A review. Fabula.

[17] Orduña R.M.D., Sant’Ana A.D.S. (2010). Climate change associated effects on grape and wine quality and production. Food Res. Int..

[18] Esteban M.A., Villanueva M.J., Lissarrague J.R. (2001). Effect of irrigation on changes in the anthocyanin composition of the skin of cv Tempranillo (*Vitis vinifera* L) grape berries during ripening. J. Sci. Food Agric..

[19] Girard B., Fukumoto L., Mazza G., Delaquis P., Ewert B. (2002). Volatile terpene constituents in maturing Gewurztraminer grapes from British Columbia. Am. J. Enol. Vitic..

[20] Schneider R., Razungles A. (2002). Effet du site, de la maturité et de l’éclairement des grappes sur la composition aromatique des baies de *Vitis vinifera* L. cv. Melon B. Bull. l’OIV.

[21] Reboredo-Rodríguez P., González-Barreiro C., Rial-Otero R., Cancho-Grande B., Simal-Gándara J. (2015). Effects of sugar concentration processes in grapes and wine aging on aroma compounds of sweet wines—A review. Crit. Rev. Food Sci. Nutr..

[22] Mccarthy M.G. (2010). The effect of transient water deficit on berry development of cv. Shiraz (*Vitis vinifera* L.). Aust. J. Grape Wine Res..

[23] Rogiers S.Y., Hatfield J.M., Jaudzems V.G., White R.G., Keller M. (2004). Grape berry cv. Shiraz epicuticular wax and transpiration during ripening and preharvest weight loss. Am. J. Enol. Vitic..

[24] Krasnow M., Matthews M., Smith R., Benz J., Weber E., Shackel K. (2008). Distinctive symptoms differentiate four common types of berry shrivel disorder in grape. Calif. Agric..

[25] Song J., Fan P.G., Wu B.H., Li S.H. (2007). Changes in soluble sugars and activities of related metabolic enzymes in grape berries during ripening and delayed harvest. Acta Hortic. Sin..

[26] Ristic R., Bindon K., Francis L.I., Herderich M.J., Iland P.G. (2010). Flavonoids and C13-norisoprenoids in Vitis vinifera L. cv. Shiraz: Relationships between grape and wine composition, wine colour and wine sensory properties. Aust. J. Grape Wine Res..

[27] Hayasaka Y., Kennedy J.A. (2003). Mass spectrometric evidence for the formation of pigmented polymers in red wine. Aust. J. Grape Wine Res..

[28] Suklje K., Zhang X., Antalick G., Clark A.C., Deloire A., Schmidtke L.M. (2016). Berry shriveling significantly alters Shiraz (*Vitis vinifera* L.) grape and wine chemical composition. J. Agric. Food Chem..

[29] Jiang B., Xi Z.M., Luo M.J., Zhang Z.W. (2013). Comparison on aroma compounds in Cabernet Sauvignon and Merlot wines from four wine grape-growing regions in China. Food Res. Int..

[30] Peng C.T., Wen Y., Tao Y.S., Lan Y.Y. (2013). Modulating the formation of Meili wine aroma by prefermentative freezing process. J. Agric. Food Chem..

[31] Genovese A., Dimaggio R., Lisanti M.T., Piombino P., Moio L. (2010). Aroma composition of red wines by different extraction methods and gas chromatography-SIM/mass spectrometry analysis. Ann. Chim. Rome.

[32] Moyano L., Zea L., Villafuerte L., Medina M. (2009). Comparison of odor-active compounds in sherry wines processed from ecologically and conventionally grown Pedro Ximenez grapes. J. Agric. Food Chem..

[33] Peinado R.A., Moreno J., Bueno J.E., Moreno J.A., Mauricio J.C. (2004). Comparative study of aromatic compounds in two young white wines subjected to pre-fermentative cryomaceration. Food Chem..

[34] Xie S., Tang Y., Wang P., Song C., Duan B., Zhang Z., Meng J. (2018). Influence of natural variation in berry size on the volatile profiles of Vitis vinifera L. cv. Merlot and Cabernet Gernischt grapes. PLoS ONE.

[35] Escudero A., Campo E., Farina L., Cacho J., Ferreira V. (2007). Analytical characterization of the aroma of five premium red wines. Insights into the role of odor families and the concept of fruitiness of wines. J. Agric. Food Chem..

[36] Forde C.G., Cox A., Williams E.R., Boss P.K. (2011). Associations between the sensory attributes and volatile composition of Cabernet Sauvignon wines and the volatile composition of the grapes used for their production. J. Agric. Food Chem..

[37] La Guerche S., Dauphin B., Pons M., Blancard D., Darriet P. (2006). Characterization of some mushroom and earthy off-odors microbially induced by the development of rot on grapes. J. Agric. Food Chem..

[38] Zhang L., Tao Y.S., Wen Y., Wang H. (2013). Aroma evaluation of young Chinese Merlot wines with denomination of origin. S. Afr. J. Enol. Vitic..

[39] Shinohara T. (2006). Gas chromatographic analysis of volatile fatty acids in wines. J. Agric. Chem..

[40] Cai J., Zhu B.Q., Wang Y.H., Lu L., Lan Y.B., Reeves M.J., Duan C.Q. (2014). Influence of pre-fermentation cold maceration treatment on aroma compounds of Cabernet Sauvignon wines fermented in different industrial scale fermenters. Food Chem..

[41] Ma T.T., Lan T., Ju Y.L., Cheng G., Que Z.L., Geng T.H., Fang Y.L., Sun X.Y. (2019). Comparison on the nutritional properties and biological activities of kiwifruit (*Actinidia*) and their different forms products: How to make kiwifruit more nutritious and functional. Food Funct..

[42] Song C.Z., Liu M.Y., Meng J.F., Shi P.B., Zhang Z.W., Xi Z.M. (2015). Influence of foliage-sprayed zinc sulfate on grape quality and wine aroma characteristics of Merlot. Eur. Food Res. Technol..

[43] OIV (2006). International Oenological Codex. http://www.oiv.int/oiv.

[44] Zhang M., Xu Q., Duan C., Qu W., Wu Y. (2007). Comparative study of aromatic compounds in young red wines from Cabernet Sauvignon, Cabernet Franc, and Cabernet Gernischet varieties in China. J. Food Sci..

[45] Kandylis P., Drouza C., Bekatorou A., Koutinas A.A. (2010). Scale-up of extremely low temperature fermentations of grape must by wheat supported yeast cells. Bioresour. Technol..

[46] Guth H. (1997). Quantitation and sensory studies of character impact odorants of different white wine varieties. J. Agric. Food Chem..

[47] Gambetta J.M., Bastian S.E.P., Cozzolino D., Jeffery D.W. (2014). Factors influencing the aroma composition of Chardonnay wines. J. Agric. Food Chem..

